# An Assessment of the Metal Removal Capability of Endemic Chilean Species

**DOI:** 10.3390/ijerph19063583

**Published:** 2022-03-17

**Authors:** Andrea Lazo, Pamela Lazo, Alejandra Urtubia, María Gabriela Lobos, Henrik K. Hansen, Claudia Gutiérrez

**Affiliations:** 1 Departamento de Ingeniería Química y Ambiental, Universidad Técnica Federico Santa María, Avenida España 1680, Valparaíso 2390123, Chile; andrea.lazo@usm.cl (A.L.); alejandra.urtubia@usm.cl (A.U.); henrik.hansen@usm.cl (H.K.H.); claudia.gutierrez@usm.cl (C.G.); 2 Instituto de Química y Bioquímica, Facultad de Ciencias, Universidad de Valparaíso, Avenida Gran Bretaña 1111, Playa Ancha, Valparaíso 2360102, Chile; gabriela.lobos@uv.cl

**Keywords:** mine tailings, endemic plants, metals

## Abstract

In Chile, there are several abandoned mine tailing impoundments near population centers that need to be remediated. In this study, the ability of *Oxalis gigantea*, *Cistanthe grandiflora,* and *Puya berteroniana* to remove Zn, Ni, and Cr from mine tailings was evaluated. The plants’ removal efficiency, bioconcentration, and translocation factors regarding these metals were determined to assess the ability of certain endemic species from Northern and Central Chile to extract or stabilize metals. After a period of seven months, the chemical analysis of plants and tailings, together with the statistical treatment of data, indicated the inability of all the species to translocate Ni, Cr, or Zn with a translocation factor lower than one. The results showed the stabilizing character of *Oxalis gigantea*, *Puya berteroniana,* and *Cistanthe grandiflora* for Zn, with a bioconcentration factor close to 1.2 in all cases, and the same ability of the latter two species for Cr, with a bioconcentration factor of 1.5 in the case of *Cistanthe grandiflora* and 1.7 for *Puya berteroniana*. Finally, a removal efficiency of 9.3% was obtained with *Cistanthe grandiflora* for Cr and 15% for Ni; values lower than 6.4% were obtained for Zn in all cases. Improvements in the process should be sought to enhance the performance of these species for the accumulation of the target metals.

## 1. Introduction

Mining activities are carried out in the northern and central zones of Chile. For a long time, due to the lack of regulations, mine tailings were stored without major environmental consideration; therefore, at present, several hills of these residues without owners are located near population centers. The tailings are made up of fine-grain material and a variable content of water, which facilitate the occurrence of chemical reactions, the dissolution of toxic substances, and the generation of sulfuric acid, with the inherent risk of soil and groundwater contamination [1,2]. This holds special relevance for Chile, where there are 173 abandoned mine tailing impoundments, and nearly 40 of these have unknown owners [3]. Many of these impoundments are located between the Region of Tarapacá and the Region of Valparaíso in the vicinity of towns, which is further worsened by the fact that Chile has no standards for soil pollution [3].

One major point of concern for environmental and public health is contamination by mining activities [1], where the negative effects of heavy metal contamination have been observed in the surrounding water, soil, and air environments of the mine sites [4,5,6]. In the case of abandoned mine tailing impoundments, negative environmental impacts have been identified, including an increase in the concentration of metals in agricultural soils, sediments, animals, human beings, and plants [7,8,9,10,11,12,13,14,15]. Among the various heavy metals, chromium (Cr) is widely present in soils, with an average concentration of 60 mg kg^−1^ [16]. Chromium is a nonessential element for plants, and it produces toxic effects in most of them at 5 mg kg^−1^, with a normal concentration in plants being less than 1 mg kg^−1^ [17]. In the case of zinc (Zn), it is an essential nutrient for plants, with a maximum value in soils for residential use of 200 mg kg^−1^ [18]. For crops, the required Zn concentration is between 15 and 20 mg kg^−1^ dry weight [19,20]. Finally, nickel (Ni) is an essential micronutrient for plants with a normal concentration between 0.05 and 10 mg kg^−1^ dry weight; at higher concentrations, it becomes toxic for plants [21]. In soils, the Dutch Environmental Standard indicates a background Ni concentration of 35 mg kg^−1^ as being acceptable [18]. The limits reported by the WHO for Zn, Cr, and Ni as permissible in plants are 0.60 mg kg^−1^, 1.30 mg kg^−1^, and 10 mg kg^−1^, respectively [22].

Phytoremediation is an alternative bioremediation technique that employs plants to recover soil or a water source without adverse effects on the environment [23]. Among the known technologies developed in recent decades, such as physicochemical and thermal processes, phytoremediation has emerged as a cost-effective remediation technology [24,25]. The main mechanisms of phytoremediation include phytoextraction and phytostabilization, both of which were assessed in the present study; the former has exhibited high efficiency and comprises a reduction in the concentrations of pollutants in the soil through their uptake by harvestable parts of the plant, while phytostabilization restricts the pollutants to the area close to the roots, halting their movement [23,26,27,28]. Plants that accumulate high concentrations of pollutants are called hyperaccumulators. In the case of heavy metals, hyperaccumulator plants contain a concentration higher than 1000 mg g^−1^ of Ni, Cu, Pb, Cr, or Co; 10,000 mg g^−1^ in the case of Zn or Mn; or up to 100 mg g^−1^ of Cd and other rare metals [29]. Until today, approximately 721 hyperaccumulator species have been discovered, most of which hyperaccumulate Ni and Zn and belong to the *Brassicaceae* family [30]. In the case of Zn, some species, mainly located in metalliferous soils in Europe, can accumulate concentrations higher than 10,000 mg kg^−1^ dry weight, most of them belonging to the *Brassicaceae* and *Crassulaceae* families [30,31]. In the case of Cr, the maximum concentration values have also been found in species of the *Brassicaceae* family [32].

The phytoremediation studies of inorganic pollutants with native flora are limited; most of the latest research has been performed with flora from Eurasia and South America [24]. The work of Chandra et al. [33] obtained translocation factors higher than 20 and 3 with *Argemone Mexicana* and *Rumex dentatus*, respectively, for Zn. The same study showed a TF = 10.45 for Ni with *Tinospora cordifolia*. In the case of *Rumex dentatus* and *Saccharum munja*, a *BCF* >1 was obtained for Zn, Cu, and Ni. Jeleni et al. [34] assessed the use of *Aristida congesta* (native) to phytoremediate alkaline mine tailings in Namibia, obtaining promising results for Cr, Ni, and Zn, and classified it as a hyperaccumulator for Cr. Another promising species is *Solanum nigrum* L., a species native to Eurasia, which has been considered as an ideal phytoremediator for several metals due to its high tolerance and easy adaptation to different conditions [35,36]. In Chile, the endemic species *Adesmia atacamensis* has been suggested as an excluder of Zn (translocation factor TF ≤ 1), where the enrichment of tailings with additives might be associated with an improvement in its phytostabilization character for Zn [37]. In another study in Chile, conducted by Lam et al. [38], *Schinus molle* (native) and *Prosopis tamarugo* (endemic) showed some features of a Zn accumulation, while *Atriplex nummularia* (introduced) was the most promising Zn accumulator.

In the present study, plants from Northern and Central Chile were used; these plant species were selected due to their adaptation to a similar area as where the tailings were sampled, their ornamental value, price, and low water requirements. The aim of this work was to assess the ability of three endemic species: *Oxalis gigantea*, *Cistanthe grandiflora,* and *Puya berteroniana* to uptake Zn, Ni, and/or Cr to remediate tailings by extraction or stabilization and, thus, complement the little information that exists on the use of Chilean endemic species for phytoremediation purposes. To evaluate them, ex situ experiments were carried out for seven months, and after the growing period, the translocation and bioconcentration factors were calculated together with their removal efficiency. 

## 2. Materials and Methods

### 2.1. Plant Species

Endemic species from 0.7 to 3 m in height that easily adapt to the climate of the area, as self-sustaining plant formations with ornamental value, were used. Moreover, the plant species were chosen considering their water requirements, predominantly for the northern and central zones of Chile. The species selected have not been used in previous studies of phytoremediation, and they are as follows:

- *Oxalis gigantea* Barnéoud, commonly called Churqui or Churco. It is a very common endemic Chilean shrub belonging to the *Oxalidaceae* family. This species can be found between the regions of Antofagasta and Coquimbo.
- *Cistanthe grandiflora*, a succulent plant, commonly called Pata de guanaco or Doquilla, is a perennial species endemic to Chile. It can be found frequently between the regions of Antofagasta and Ñuble, and it belongs to the *Portulacaceae* family.
- *Puya berteroniana* is an endemic Chilean shrub from the *Bromeliaceae* family. This species grows between the regions of Coquimbo and Maule. It is commonly called Chagual or Magüey, and it has excellent ornamental value.

*Oxalis gigantea* Barnéoud is hardy to the United States Department of Agriculture (USDA) Zones 10 and 11, while *Cistanthe grandiflora* and *Puya berteroniana* are hardy to USDA Zone 9 [39,40].

**Figure 1:**
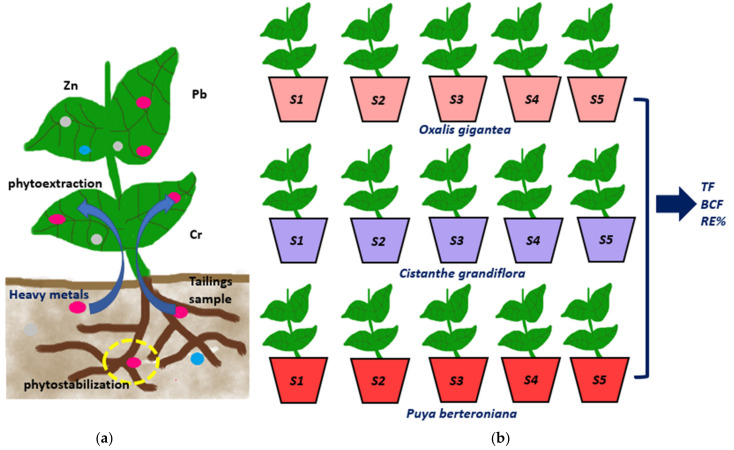
(**a**) Schematic representation of phytoextraction and phytostabilization; (**b**) experimental setup for phytoremediation pot experiments: five triplicates for each species. From these experiments, the translocation factor, bioconcentration factor, and removal efficiency were calculated.

All plants were acquired from a garden center of native and endemic flora. The experimental setup is shown in Figure 1.

The plants were transplanted to pots with 1440 g of tailings with an initial height of 10 cm. The depth of the tailings in the pot was 20 cm in all cases. The nutrients were incorporated through periodic application of a foliar organic stimulant (made of marine algae *Ascophyllum nodosum*) with the following concentrations: N: 0.1% *w*/*w*, K: 3.0% *w*/*w*, As: <0.5 mg kg^−1^, Cd: <0.5 mg kg^−1^, Pb: <1 mg kg^−1^, and Hg: <0.5 mg kg^−1^. Tap water and foliar stimulant were provided weekly in spray form; no leaching was collected from pots as they did not have holes.

The experiments were carried out ex situ for seven months, and the plants were maintained outdoors at the Universidad Técnica Federico Santa María, where climatic conditions such as Cabildo prevail (the mine tailing impoundment location).

### 2.2. Tailing Samples

Paste tailing samples from the copper mine held by Compañía Minera Las Cenizas in Cabildo, Region of Valparaíso, Chile, with coordinates 32°28′16.1″ S, 71°05′00.2″ W, were used in the present study. This mining company processed copper sulfide and oxide minerals. 

After sampling, the tailings were dried in a laboratory oven at 105 °C to achieve a constant mass; then, they were ground in a hammer mill, sieved through a mesh (ASTM No. 18), and homogenized. The pH was measured using the U.S. Environmental Protection Agency Method 9045D [41]. The content of the oxidized compounds was obtained from Servicio Nacional de Geología y Minería de Chile [3]. The elemental composition was measured with inductively coupled atomic emission spectroscopy (ICP-OES), Perkin Elmer ICP Optima 2000DV, with a detection limit between 0.005 and 0.01 ppm, depending upon the element. Table 1 presents the pH, granulometric characteristics, and composition of the tailings. 

### 2.3. Sample Preparation and ICP-OES Measurements

After the growth period, the plants were carefully removed from the pots; then, the roots were separated from the stems and leaves, called the aerial parts in the remainder of the paper, and both parts were washed with abundant tap water, distilled water, and finally deionized water to eliminate all tailing remnants. The excess water was removed with paper tissue. Stems and leaves were separated from roots, and each part was prepared separately. The parts of the plants were divided into small pieces, placed in waxed paper envelopes, and dried in a laboratory oven at 45 °C until they achieved a constant mass. Finally, they were pulverized and homogenized, and representative samples were taken using the quartering method. Each plant was individually prepared. The aerial parts and roots of each plant were digested separately using 0.200 g dry weight. Each sample was placed in a Teflon vial with 2 mL of concentrated H_2_O_2_ and 8 mL of concentrated HNO_3_. A 4-h predigestion was carried out before the digestion in an Ethos Easy microwave. The program used in the microwave consisted of three stages: (1) a linear increase from room temperature to 180 °C for 10 min, (2) a plateau for 10 min, and (3) a linear decrease in temperature for 10 min. When the samples cooled, they were placed in a 25-mL volumetric flask and filled with deionized water to the calibration line. 

In the case of the tailings, the total mass of each pot was dried at 105 °C until a constant mass was achieved, followed by sieving through a mesh and homogenization. The quartering method was used to take representative samples. The digestion procedure was the same as for the plants but using a 0.06 g dry weight.

Five plants from each species were analyzed. From each plant, the total mass of the stem and leaves was dried, ground, and homogenized. The quartering method was used to take three representative samples. In the case of the roots, the procedure was the same. All samples were measured in triplicate.

The concentrations of the elements under study were measured by inductively coupled atomic emission spectroscopy (ICP-OES).

### 2.4. Theory/Calculations

To assess the phytoremediation potential, translocation and bioconcentration factors were used. The translocation factor (TF) allows for the evaluation of the ability of the plant to translocate contaminants from roots to stems and leaves, such that when the plant presents a TF > 1 it can be considered an accumulator, while a TF ≤ 1 is indicative that the plant is an excluder. In the same way, the bioconcentration factor (*BCF*) gives information about the plant’s ability to accumulate the target element, and a *BCF* >1 indicates the potential success of the phytoremediation technique. Those species that stabilize a metal present a *BCF* ≥ 1 and a TF ≤ 1 [42,43,44], and they are potentially useful for phytostabilization. Moreover, the removal efficiency (*RE*) allows us to assess the decrease in the concentration of the target element in the tailings.

TF values were calculated for different species and metals with Equation (1).


(1) $$\mathrm{BCF}=\frac{Metal\;concentration\;in\;stern\;and\;leaves}{Metal\;concentration\;in\;roots}$$

The bioconcentration factor (*BCF*) was obtained for the roots using Equation (2) [39,44,45,46,47].


(2) $$\mathrm{BCF}=\frac{Metal\;concentration\;in\;roots}{Initial\;concentration\;of\;metal\;in\;tailing}$$

The removal efficiency (*RE*) was calculated with Equation (3),

(3) $$\mathrm{RE}=\frac{\left(C_{i}-C_{f}\right)}{C_{i}}\times 100\%$$

where *C_i_* and *C_f_* were the initial and final concentrations of the element in the tailings.

To compare the metal concentration in the roots or aerial parts, a one-way analysis of variance (ANOVA) was performed with a significance level of *p* < 0.05 for the comparison of means. Then, a Tukey test was carried out to compare the different species.

## 3. Results

### 3.1. Concentration of Metals in Tailings

After seven months, the tailings and plants were analyzed. The initial and final concentrations of elements in the tailings are shown in Table 2.

### 3.2. Concentration of Metals in Plants and Control Samples

Control samples consisted of the species studied planted in pots (without holes) with unpolluted soil. They were analyzed before and after the growth period. The concentrations of Cr, Ni, and Zn are presented in Table 3.

The initial concentrations of Cr, Ni, and Zn were measured in the roots and aerial parts of the plants before the beginning of the experiments. The values of the mean concentrations and standard deviation (SD) are presented in Table 4.

As observed in Table 4, the plants had no detectable Cr concentrations, and the levels of Ni and Zn were normal for plants.

**Figure 2:**
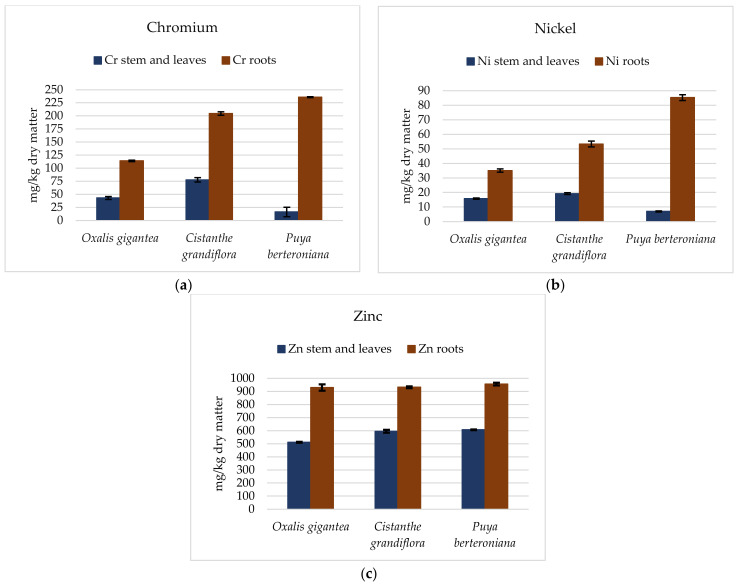
Metal content in studied plant species. Values are mean ± standard deviation. (**a**) Cr in stems and leaves, and roots; (**b**) Ni in stems and leaves, and roots; (**c**) Zn in stems and leaves, and roots.

The final concentration of elements was determined in the roots and aerial parts. The results, presented as the mean concentrations and standard deviation, are presented in Figure 2. No plants died before the end of the experiments. 

In the comparison between species, for aerial parts, one-way ANOVA and the Tukey test indicated statistically significant differences between species in all cases. In the case of the root analysis, no statistically significant differences were obtained for Zn. The results of the Tukey test are presented in Table 5.

It can be observed from Figure 2 that the concentration of Cr in the aerial parts was greater than or close to 80 mg kg^−1^ in all samples of *Cistanthe grandiflora*, decreasing by half in the case of *Oxalis gigantea*, and close to 20 mg kg^−1^ in the case of *Puya berteroniana*; therefore, all these values far exceeded the concentration of 5 mg kg^−1^, which has been indicated as the value that marks the beginning of toxicity [17]. The ability to accumulate Cr in the aerial parts decreased by 50% successively as follows: *Cistanthe grandiflora* > *Oxalis gigantea* > *Puya berteroniana*. Additionally, Cr in the roots largely exceeded the concentration in the aerial parts, where *Puya berteroniana* accumulated a concentration close to 240 mg kg^−1^, followed by *Cistanthe grandiflora* with a mean concentration of 200 mg kg^−1^.

Nickel in the roots and aerial parts showed the same trend as Cr in the different species; thus, the greatest accumulation in the aerial parts was 20 mg kg^−1^ for *Cistanthe grandiflora* and 86 mg kg^−1^ in *Puya berteroniana* in the case of the roots. It should be noted that all concentrations determined in plants in the case of Ni were over the lower limit of toxicity for Ni (12.4 mg kg^−1^) [21].

In the case of Zn, the accumulated concentrations in roots did display not significant differences. In the case of the aerial parts, significant differences were presented between *Oxalis Gigantea*/*Puya berteroniana* and *Oxalis gigantea*/*Cistanthe grandiflora*. We observed a mean concentration in roots of around 950 mg kg^−1^, while in the aerial parts, *Cistanthe grandiflora* and *Puya berteroniana* accumulated 600 mg kg^−1^ of Zn. *Oxalis gigantea* presented a lower concentration of 510 mg kg^−1^. Both values surpassed the normal value of Zn in plants (crops).

### 3.3. Phytoremediation Factors and Removal Efficiency

**Figure 3:**
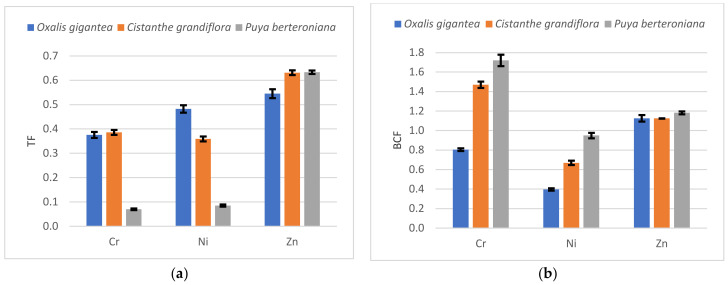
Phytoremediation factors ± standard deviation for Ni, Zn, and Cr in all studied species. (**a**) Translocation factor; (**b**) bioconcentration factor.

To analyze the ability of the species to extract or stabilize the target metals, TF and *BCF* were calculated after the growth period. The results are presented in Figure 3.

Based on Figure 3, it can be observed that none of the species presented a TF > 1; therefore, all lack the ability to translocate Ni, Zn, or Cr from the roots to the aerial parts. However, the three studied species, *Oxalis gigantea*, *Cistanthe grandiflora,* and *Puya berteroniana*, can stabilize Zn with a *BCF* higher than 1, and the latter two have the potential for Cr phytostabilization with a *BCF* of around 1.4 and 1.7, respectively.

The species *Puya berteroniana* presented better features for Cr, Ni, and Zn stabilization followed by *Cisthante grandiflora*.

**Figure 4:**
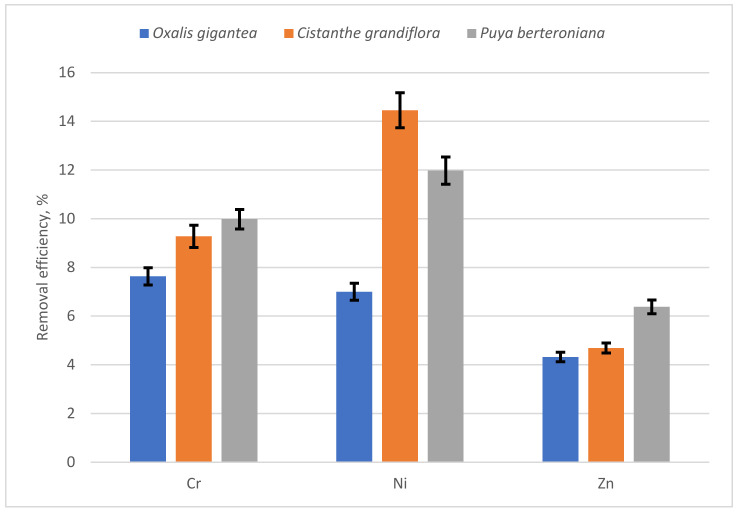
Removal efficiency ± standard deviation for Ni, Zn, and Cr for all studied species.

Figure 4 shows the removal efficiency for Cr, Ni, and Zn for each species. In the case of Cr and Zn, the highest removal efficiency was obtained with *Puya berteroniana* (10% and 6.4%, respectively), while for Ni, the highest efficiency was achieved with *Cistanthe grandiflora* (15%). The lowest efficiencies were obtained with *Oxalis gigantea* for all studied metals.

Among the species that were studied, *Cistanthe grandiflora* and *Puya berteroniana* were shown to be chromium and nickel stabilizers, with a removal efficiency higher than 10%.

## 4. Discussion

The mining industry in Chile is highly developed; however, for many years, it did not take responsibility for its environmental effects. In Chile, there is no legislation relative to pollutants in soils; for that reason, the Dutch Environmental Standard was used for comparison [48], and according to this, the intervention values for Zn and Ni are 720 and 100 mg kg^−1^ dry weight. They are not indicated for Cr [48], but Srivastava et al. [16] indicated an acceptable level in soils for human health and the environment of 64 mg kg^−1^. The lack of regulations produced a high number of abandoned tailings in Northern and Central Chile. The problems generated by these environmental liabilities are deepening due to the limited resources of the municipalities that must take charge of these residues, which imposes the need to develop an economic, easy, and environmentally friendly technology to remediate the tailings. In 2019, the Chilean government detected 37 abandoned tailing impoundments with a potential risk for nearby towns, which constitutes less than 25% of the total abandoned impoundments and 5% of the total impoundments [3,49].

Moreover, tailings are not a nutritive medium, they have a poor water-holding capacity, and they are normally exposed to severe environmental conditions [45].

It is a well-known fact that using native or endemic species increases the chance that a phytoremediation process will be successfully implemented. This success lies in the establishment of a plant community, of species that adapt easily to local conditions, with the ability to remove the pollutants of interest [15,24]. Plant stress is increased when plants are exposed to a lack of nutrients and organic matter or large variations in temperature [15,45]. For the above, to carry out a phytoremediation process in tailings successfully, the use of native or endemic flora is considered essential. This research focused on the assessment of the ability of certain endemic species of the zone where mining activities are carried out to remove Cr, Zn, and Ni, considering that the most important pollutants in tailings are Cu, Cr, Ni, Zn, Pb, As, Cd, and Hg [50].

The selection of the species was based on their fast growth, good ornamental value, low price, and low water requirements. The latter is essential due to the existing water scarcity in the main mining areas and the drought experienced in the country.

### 4.1. Presence of Metals in the Original Tailings

As can be seen from Table 2, the initial concentration of Zn in the tailings exceeded the values indicated by the Dutch Environmental Standards. In the case of Ni, the initial level was very close to the intervention value, and, finally, levels of Cr in the original samples did not only exceed the indicated value for intervention but doubled it. Unfortunately, in Chile, there are several abandoned tailings close to rivers and population centers; nevertheless, it is not possible to label this pollution, because there is no legislation regarding soil pollution [3].

The initial concentrations of Ni and Zn were higher in the roots than in the aerial parts in the case of *Oxalis gigantea* in all samples; however, in the case of *Cistanthe grandiflora* and *Puya berteroniana,* the trend was reversed. Chromium was below the detection limit of the instrument in most samples. All concentrations in the plants were within normal values.

Control samples were maintained over the complete period of growth, and they were analyzed before and after this period, showing no significant differences in Cr, Ni, and Zn. The above indicates that the increase in the final concentrations of the target elements is only due to the effect of the elements contained in the tailings.

### 4.2. Efficiency of the Phytoremediation Process

There have been a few studies carried out with tailings in Chile under climatic conditions similar to those found in the central and northern zones. One of these studies was carried out by Lam et al. [39], who studied the phytoremediation of Cu, Fe, Mn, Pb, Zn, and Cd. From this study, carried out over one year of growth, they observed a reduced performance by three species (*Prosopis tamarugo*, *Schinus molle,* and *Atriplex nummularia*) in tailings without amendments, obtaining, in all cases, an improvement when a mixture with CaCO_3_ + compost or CaCO_3_ + compost + *mycorrhizal fungi* was used. None of these species exhibited the ability to stabilize the metals, but the last two showed high translocation factors for Zn, with a removal efficiency of 40 and 60%, respectively. The species used by Lam et al. [39] can reach several meters in height; the first two are trees, and the latter is a large shrub. In the case of the present study, all species can reach three meters as a maximum height; they showed a slight stabilizing character for Zn with removal efficiencies lower than 10%. None of them presented a translocation factor higher than 1, evidencing their poor performance regarding the phytoextraction of Zn and the rest of the target elements.

In the case of Ni, there is a lack of studies with endemic Chilean species. In other countries, there are native species with the ability to phytoremediate Ni, with a high capacity to bioaccumulate it. In the work of Wu et al. [15], several native Chinese plants were assessed for soil phytoremediation, and the authors obtained a TF > 1 with *Polygonum capitanum* and promising results for the phytoextraction of Ni with *Miscanthus floridulus*, Conyza canadensis, and *Rubus setchuenensis*. The concentration of Ni accumulated in the roots and aerial parts was similar to that obtained in this study. Jeleni et al. [33] used *Aristida congesta*, a native grass of Namibia, to phytoremediate tailings, obtaining a mean concentration in roots of 50 mg kg^−1^ and in aerial parts close to 25 mg kg^−1^. The pH of the tailings used by Jeleni et al. [33] was 1.5 points more alkaline than the tailings in the present study. Comparing the concentrations, *Puya berteroniana* achieved a higher accumulation in the roots; however, the performance of all species was inferior in the case of the aerial parts. The removal efficiency for Ni with *Cistanthe grandiflora* was 15%, and the efficiencies for the other two species were very close to this value. However, only *Puya berteroniana* showed a *BCF* close to 1, and none of the species studied presented a TF higher than 1 for Ni.

For Cr, promising results were obtained using *Puya berteroniana* and *Cistanthe grandiflora* to stabilize this metal, but the removal efficiencies were close to 10% for seven months of growth. Considering the limited budget of the municipalities in charge of these liabilities, the extraction and incineration of the plants are a very unlikely alternative; thus, stabilization becomes an economic alternative to reduce the mobility of the metal and the possibility of leaching. Similar studies with Chilean native or endemic flora were not found; however, Manikandan et al. [51] screened several native species for the phytoremediation of Cr from soil contaminated by tannery effluent. The species *Acacia auriculiformis* was cataloged as a hyperaccumulator of Cr. In addition, other species, *Dalbergia sisso* and *Thespesia populnea*, concentrated large quantities of this element without falling into this category. Translocation and bioconcentration factors were not calculated; hence, it is not possible to compare these with the present study. Based on the concentrations in roots, a value of 200 mg kg^−1^ was exceeded with *Puya berteroniana* and *Cistanthe grandiflora*, but very low concentrations were achieved in the aerial parts < 20 and 75 mg kg^−1^, respectively. The work of Manikandan et al. [51] indicated concentrations in the roots close to 10,000 mg kg^−1^ and 5000 mg kg^−1^ in the aerial parts with *Acacia auriculiformis* and 36,000 mg kg^−1^ in the roots and 1500 mg kg^−1^ in the aerial parts in the case of *Albizzia lebbeck*. However, only a few Cr hyperaccumulator species have been found up to now. Other species recognized for the phytoremediation of Cr are *Plunchea indica*, *Cynodon dactylon*, *Phragmites australis*, *Typha angustifolia*, *Pterocarpus indicus,* and *Jatropha curcas* [52]. No endemic Chilean species has been cataloged as a hyperaccumulator of Cr.

All the removal efficiencies were calculated using the mean value; however, in the case of Cr and Zn, some results were inconclusive. The analysis of the results and the discussion was mainly focused on the chemical analysis of the aerial parts and roots. Technically, a mass balance would solve this problem; however, in this case, due to the fine roots of the plants, the complete biomass could not be fully recovered from the tailings. Among the species studied, *Puya berteroniana* presented the best performance to stabilize Cr and Zn. Regarding the latter, future research will be focused on the use of nanoparticles and chemical chelators to improve the mobility of metallic ions and/or the ability of these species to concentrate the elements of interest; moreover, the use of different mixtures of amendments and compost will be explored, and the rate of accumulation of each metal and each species will be determined.

## 5. Conclusions

In this study, the ability of three plant species—*Cistanthe grandiflora*, *Puya beteroniana,* and *Oxalis gigantea*—to extract or stabilize Cr, Ni, and Zn was assessed. These endemic Chilean species belong to families not traditionally considered in the phytoremediation of contaminated substrates, and they were selected based on their adaptation to extreme conditions, low water requirements, ornamental value, availability, and low cost.

Among the species studied, *Cistanthe grandiflora* and *Puya berteroniana* can accumulate Cr and Zn but not translocate them, showing their potential to phytostabilize these elements, obtaining a bioconcentration factor for Zn close to 1.2 for both species, 1.5 in the case of *Cistanthe grandiflora* for Cr, and 1.7 for *Puya berteroniana* for the same metal. *Oxalis gigantea* only showed the ability to concentrate Zn in the roots, with a *BCF* close to 1.2, similar to the value obtained with the other species. In the case of Ni, its bioconcentration factor was under 1 in all species.

Promising results were obtained only in the case of Cr and Zn with *Puya berteroniana* and *Cistanthe grandiflora*, but their removal efficiencies were low, not exceeding 10.0% and 6.4%, respectively. Additional work should be conducted to improve the performance of these species in order to make them a real alternative for the phytostabilization of tailings.

## Figures and Tables

**Table 1:** pH value, granulometric composition, and composition of the tailings.

Parameter	Value ± Standard Deviation
Specific gravity	2.78 ± 0.25
pH	7.30 ± 0.10
Solid concentration (weight %)	82.00 ± 1
Granulometry d_50_ micrometers	0.046 ± 0.001
Electric conductivity (dS m^−1^ at 25 °C)	32.20 ± 0.10
Cu mg kg^−1^ dry weight	1582.22 ± 78.31
Mo mg kg^−1^ dry weight	3.86 ± 0.17
Pb mg kg^−1^ dry weight	228.15 ± 2.79
Zn mg kg^−1^ dry weight	869.80 ± 31.54
Ni mg kg^−1^ dry weight	94.64 ± 2.57
Cr mg kg^−1^ dry weight	154.63 ± 5.41
Cd mg kg^−1^ dry weight	Under detection limit
Calcium percentage as oxide, CaO% *w*/*w*	9.92 ± 0.32
Magnesium percentage as oxide, MgO% *w*/*w*	5.56 ± 0.27
Manganese percentage as oxide, MnO% *w*/*w*	0.26 ± 0.01
Sodium percentage as oxide, Na_2_O% *w*/*w*	2.63 ± 0.11
Potassium percentage as oxide, K_2_O% *w*/*w*	2.24 ± 0.12
Phosphorous percentage as oxide, P_2_O_5_% *w*/*w*	0.23 ± 0.01

**Table 2:** Initial and final concentrations of elements in tailings ± standard deviation.

Element	Concentration mg kg^−1^ Dry Weight ± Standard Deviation			
	Initial Tailings	Final Tailings		
		*Oxalis gigantea*	*Cistanthe grandiflora*	*Puya berteroniana*
Zn	869.80 ± 31.54	832.17 ± 32.67	828.96 ± 25.67	803.88 ± 37.89
Ni	94.64 ± 2.57	87.98 ± 3.32	80.93 ± 2.89	83.27 ± 4.06
Cr	154.63 ± 5.41	142.79 ± 6.52	140.25 ± 5.98	141.83 ± 4.87

**Table 3:** The initial and final concentrations of Cr, Ni, and Zn in the control samples ± standard deviation.

Element	Concentration mg kg^−1^ Dry Weight ± Standard Deviation		
	*Oxalis gigantea*	*Cistanthe grandiflora*	*Puya berteroniana*
Cr stems and leaves t = 0	<d.l	<d.l	<d.l
Cr stems and leaves t = t_final_	0.01 ± 0.00	0.01 ± 0.00	<d.l
Cr roots t = 0	0.01 ± 0.00	<d.l	<d.l
Cr roots t = t_final_	0.01 ± 0.00	<d.l	<d.l
Ni stems and leaves t = 0	1.85 ± 0.08	6.12 ± 0.28	5.76 ± 0.08
Ni stems and leaves t = t_final_	2.01 ± 0.06	6.25 ± 0.19	6.76 ± 0.12
Ni roots t = 0	2.55 ± 0.10	4.14 ± 0.52	1.97 ± 0.05
Ni roots t = t_final_	2.85 ± 0.07	4.56 ± 0.47	1.81 ± 0.05
Zn stems and leaves t = 0	13.39 ± 0.45	28.79 ± 0.60	24.10 ± 0.95
Zn stems and leaves t = t_final_	13.25 ± 0.55	32.55 ± 0.42	23.55 ± 0.88
Zn roots t = 0	16.82 ± 0.50	16.12 ± 0.09	17.96 ± 0.88
Zn roots t = t_final_	17.76 ± 0.61	17.65 ± 0.08	18.33 ± 0.95

<d.l: below the detection limit.

**Table 4:** The initial concentrations of Cr, Ni, and Zn in plants ± standard deviation.

Element	Concentration mg kg^−1^ Dry Weight ± Standard Deviation		
	*Oxalis gigantea*	*Cistanthe grandiflora*	*Puya berteroniana*
Cr stems and leaves	<d.l	<d.l	<d.l
Cr roots	0.14 ± 0.00	<d.l	<d.l
Ni stems and leaves	1.98 ± 0.09	6.32 ± 0.30	5.53 ± 0.06
Ni roots	2.86 ± 0.09	3.80 ± 0.45	1.97 ± 0.07
Zn stems and leaves	13.97 ± 0.50	28.24 ± 0.65	24.75 ± 1.10
Zn roots	16.79 ± 0.47	16.44 ± 0.09	18.25 ± 0.90

<d.l: below the detection limit.

**Table 5:** ANOVA and Tukey test results for Cr, Ni, and Zn.

Cr Stems and Leaves	Sum of Squares	*df*	Mean Square	*F*	*p*-Value	*F. crit*	
Between groups	9457.54	2	4728.77	1115.96	2.34 × 10^−14^	3.89	
Within groups	50.87	12	4.24				
Total	9508.41	14					
		mean	Std. error	*q-stat*	lower	upper	*p*-value
*Oxalis gigantea*	*Puya berteroniana*	34.62	0.92	37.60	31.15	38.10	<0.05
*Oxalis gigantea*	*Cistanthe grandiflora*	26.71	0.92	29.01	23.24	30.19	<0.05
*Puya berteroniana*	*Cistanthe grandiflora*	61.34	0.92	66.62	57.86	64.81	<0.05
Cr roots	Sum of groups	*df*	Mean square	*F*	*p*-value	*F. crit*	
Between groups	39,951.43	2	19,975.71	338.42	1.44 × 10^−12^	3.89	
Within groups	429.27	12	35.77				
Total	40,380,70						
		mean	Std. error	*q-stat*	lower	upper	*p*-value
*Oxalis gigantea*	*Puya berteroniana*	90.47	2.67	33.83	80.36	100.57	<0.05
*Oxalis gigantea*	*Cistanthe grandiflora*	121.70	2.67	45.97	111.60	131.79	<0.05
*Puya berteroniana*	*Cistanthe grandiflora*	31.22	2.67	11.67	21.13	41.31	<0.05
Ni stems and leaves	Sum of squares	*df*	Mean square	*F*	*p*-value	*F. crit*	
Between groups	411.61	2	205.81	819.49	1.47 × 10^−34^	3.89	
Within groups	3.01	12	0.25				
Total		14					
		Mean	Std. error	*q-stat*	Lower	Upper	*p*-value
*Oxalis gigantea*	*Puya berteroniana*	2.75	0.22	12.27	1.90	3.60	<0.05
*Oxalis gigantea*	*Cistanthe grandiflora*	9.48	0.22	42.30	8.63	10.32	<0.05
*Puya berteroniana*	*Cistanthe grandiflora*	12.23	0.22	54.57	11.38	13.07	<0.05
Ni roots	Sum of squares	*df*	Mean square	*F*	*p*-value	*F. crit*	
Between groups	4459.05	2	3229.53	1061.87	3.15 × 10^−14^	3.89	
Within groups	36.50	12	3.04				
Total	6495.55	14					
		Mean	Std. error	*q-stat*	Lower	Upper	*p*-value
*Oxalis gigantea*	*Puya berteroniana*	18.21	0.78	23.36	15.27	21.16	<0.05
*Oxalis gigantea*	*Cistanthe grandiflora*	50.20	0.78	64.37	47.26	53.14	<0.05
*Puya berteroniana*	*Cistanthe grandiflora*	31.99	0.78	41.01	29.04	34.93	<0.05
Zn stems and leaves	Sum of squares	*df*	Mean square	*F*	*p*-value	*F. crit*	
Between groups	9457.54	2	4728.77	1115.60	2.34 × 10^−14^	3.89	
Within groups	50.86	12	4.24				
Total	9508.41	14					
		Mean	Std. error	*q-stat*	Lower	Upper	*p*-value
*Oxalis gigantea*	*Puya berteroniana*	83.87	3.16	26.52	71.94	95.79	<0.05
*Oxalis gigantea*	*Cistanthe grandiflora*	95.45	3.16	30.19	83.52	107.238	<0.05
*Puya berteroniana*	*Cistanthe grandiflora*	11.59	3.16	3.67	−0.34	23.52	0.06
Zn roots	Sum of squares	*df*	Mean square	*F*	*p*-value	*F. crit*	
Between groups	2118.66	2	1059.33	4.41	0.04	3.89	
Within groups	2880.03	12	240.00				
Total	4998.69	14					
		Mean	Std. error	*q-stat*	Lower	Upper	*p*-value
*Oxalis gigantea*	*Puya berteroniana*	2.31	6.93	0.33	−23.83	28.44	0.97
*Oxalis gigantea*	*Cistanthe grandiflora*	26.28	6.93	3.79	0.14	52.43	0.05
*Puya berteroniana*	*Cistanthe grandiflora*	23.98	6.93	3.46	−2.16	50.12	0.07

## Data Availability

Not applicable.

## References

[1] Karaca O., Cameselle C., Reddy K.R. (2018). Mine tailing disposal sites: Contamination problems, remedial options and phytocaps for sustainable remediation. Rev. Environ. Sci. Biotechnol..

[2] Lam E.J., Zetola V., Ramírez Y., Montofré Í.L., Pereira F. (2020). Making Paving Stones from Copper Mine Tailings as Aggregates. Int. J. Environ. Res. Public Health.

[3] Sernageomin Servicio Nacional de Geología y Minería (Chile). https://www.sernageomin.cl/datos-publicos-deposito-de-relaves/.

[4] Uugwanga M., Kgabi N. (2020). Assessment of Metals Pollution in Sediments and Tailings of Klein Aub and Oamites Mine Sites, Namibia. Environ. Adv..

[5] Radziemska M., Vaverková M.D., Baryla A. (2017). Phytostabilization—Management Strategy for Stabilizing Trace Elements in Contaminated Soils. Int. J. Environ. Res. Public Health.

[6] Pourret O., Bollinger J.-C., Hursthouse A. (2021). Heavy metal: A misused term. Acta Geochim..

[7] Wang Z., Bao J., Wang T., Moryani H.T., Kang W., Zheng J., Zhan C., Xiao W. (2021). Hazardous Heavy Metals Accumulation and Health Risk Assessment of Different Vegetable Species in Contaminated Soils from a Typical Mining City, Central China. Int. J. Environ. Res. Public Health.

[8] Sun Z., Xie X., Wang P., Hu Y., Cheng H. (2018). Heavy metal pollution caused by small-scale metal ore mining activities: A case study from a polymetallic mine in South China. Sci. Total Environ..

[9] Luo C., Routh J., Dario M., Sarkar S., Wei L., Luo D., Liu Y. (2020). Distribution and mobilization of heavy metals at an acid mine drainage affected region in South China, a post-remediation study. Sci. Total Environ..

[10] Akoto R., Anning A.K. (2021). Heavy metal enrichment and potential ecological risks from different solid mine wastes at a mine site in Ghana. Environ. Adv..

[11] Liu X., Chen S., Yan X., Liang T., Yang X., El-Naggar A., Liu J., Chen H. (2021). Evaluation of potential ecological risks in potential toxic elements contaminated agricultural soils: Correlations between soil contamination and polymetallic mining activity. J. Environ. Manag..

[12] Wang P., Sun Z., Hu Y., Cheng H. (2019). Leaching of heavy metals from abandoned mine tailings brought by precipitation and the associated environmental impact. Sci. Total Environ..

[13] Muthusamy L., Rajendran M., Ramamoorthy K., Narayanan M., Kandasamy S., Kumar V., Shah M.P., Kumar Shahi S. (2022). 11-Phytostabilization of metal mine tailings—A green remediation technology. Phytoremediation Technology for the Removal of Heavy Metals and Other Contaminants from Soil and Water.

[14] Urrutia C., Mansilla-Yañez R., Jeison D. (2019). Bioremoval of heavy metals from metal mine tailings water using microalgae biomass. Algal Res..

[15] Wu B., Peng H., Sheng M., Luo H., Wang X., Zhang R., Xu F., Xu H. (2021). Evaluation of phytoremediation potential of native dominant plants and spatial distribution of heavy metals in an abandoned mining area in Southwest China. Ecotoxicol. Environ. Saf..

[16] Srivastava D., Tiwari M., Dutta P., Singh P., Chawda K., Kumari M., Chakrabarty D. (2021). Chromium Stress in Plants: Toxicity, Tolerance and Phytoremediation. Sustainability.

[17] Oliveira H. (2012). Chromium as an Environmental Pollutant: Insights on Induced Plant Toxicity. J. Bot..

[18] Rijkswaterstaat Ministry of Infrastructure and the Environment (2014). Into Dutch Soils. www.rijkswaterstaat.nl/en.

[19] Broadley M.R., White P.J., Hammond J.P., Zelko I., Lux A. (2007). Zinc in plants. New Phytol..

[20] Sadeghzadeh B. (2013). A review of zinc nutrition and plant breeding. J. Soil Sci. Plant Nutr..

[21] Hassan M.U., Chattha M.U., Khan I., Chattha M.B., Aamer M., Nawaz M., Ali A., Khan M.A.U., Khan T.A. (2019). Nickel toxicity in plants: Reasons, toxic effects, tolerance mechanisms, and remediation possibilities—A review. Environ. Sci. Pollut. Res..

[22] WHO (1996). Permissible Limits of Heavy Metals in Soil and Plants.

[23] Yan A., Wang Y., Ngin T.S., Lokman M.Y.M., Subhadip G., Zhong C. (2020). Phytoremediation: A Promising Approach for Revegetation of Heavy Metal-Polluted Lan. Front. Plant Sci..

[24] Futughe A.E., Purchase D., Jones H., Shmaefsky B. (2020). Phytoremediation Using Native Plants. Phytoremediation. Concepts and Strategies in Plant Sciences.

[25] Nedjimi B. (2021). Phytoremediation: A sustainable environmental technology for heavy metals decontamination. SN Appl. Sci..

[26] Policarpo F.C., Policarpo F.M., Severino M., De Melo Nunes N.A., Ahmad Bhat R., Policarpo M.F., Hamid Dar G., Hakeem K. (2022). Chapter 3—Mechanisms of phytoremediation. Phytoremediation Biotechnological Strategies for Promoting Invigorating Environs.

[27] Ashraf S., Ali W., Zahir Z.A., Ashraf S., Asghar H.N. (2019). Phytoremediation: Environmentally sustainable way for reclamation of heavy metal polluted soils. Ecotoxicol. Environ. Saf..

[28] Yadav R., Singh S., Kumar A., Singh A.N., Kathi S., Devipriya S., Thamaraiselvi K. (2022). Chapter 15—Phytoremediation: A wonderful cost-effective tool. Advances in Environmental Pollution Research, Cost Effective Technologies for Solid Waste and Wastewater Treatment.

[29] Bilal T., Malik B., Tahir I., Kumar M., Varma A., Rehman R., Hakeem K., Sabir M., Ozturkt M., Mermut A. (2015). Chapter 5—Phytoremediation: An Eco-Friendly Green Technology for Pollution Prevention, Control and Remediation. Soil Remediation and Plants, Prospects and Challenges.

[30] Reeves R.D., Baker A.J.M., Jaffré T., Erskine P.D., Echevarria G., Van der Ent A. (2017). A global database for plants that hyper accumulate metal and metalloid trace elements. New Phytol..

[31] Balafrej H., Bogusz D., Triqui Z.-E.A., Guedira A., Bendaou N., Smouni A., Fahr M. (2020). Zinc Hyperaccumulation in Plants: A Review. Plants.

[32] Zeremski T., Ranđelović D., Jakovljević K., Marjanović Jeromela A., Milić S. (2021). Brassica Species in Phytoextractions: Real Potentials and Challenges. Plants.

[33] Chandra R., Kumar V., Tripathi S., Sharma P. (2018). Heavy metal phytoextraction potential of native weeds and grasses from endocrine-disrupting chemicals rich complex distillery sludge and their histological observations during in-situ phytoremediation. Ecol. Eng..

[34] Jeleni M.N., Gumbo J.R., Muzerengi C., Dacosta F.A. (2012). An Assessment of Toxic Metals in Soda Mine Tailing and a Native Grass: A Case Study of an Abandoned Nyala Magnesite Mine, Limpopo, South Africa. WIT Trans. Ecol. Environ..

[35] Ji P., Huang X., Jiang Y., Zhao H. (2020). Potential of enhancing the phytoremediation efficiency of *Solanum nigrum* L. by earthworms. Int. J. Phytoremediat..

[36] Cailian Y., Xianlong P., Hong Y., Xiaoxia L., Zhenhua Z., Tingliang Y. (2015). Phytoremediation Ability of *Solanum nigrum* L. to Cd-Contaminated Soils with High Levels of Cu, Zn and Pb. Water Air Soil Pollut..

[37] Lam E.J., Keith B.F., Montofré Í.L., Gálvez M.E. (2018). Copper Uptake by *Adesmia atacamensis* in a Mine Tailing in an Arid Environment. Air Soil Water Res..

[38] Lam J., Cánovas M., Gálvez M.E., Montofré I.L., Keith B.F., Faz Á. (2017). Evaluation of the phytoremediation potential of native plants growing on a copper mine tailing in northern Chile. J. Geochem. Explor..

[39] Chileflora. http://www.chileflora.com/Florachilena/FloraSpanish/PIC_NORTHERN_PLANTS_0.php.

[40] Lazo P., Lazo A. (2020). Assessment of Native and Endemic Chilean Plants for Removal of Cu, Mo and Pb from Mine Tailings. Minerals.

[41] U.S. Environmental Protection Agency SW-846 Test Method 9045D: Soil and Waste pH. https://www.epa.gov/hw-sw846/sw-846-test-method-9045d-soil-and-waste-ph.

[42] Mishra T., Pandey V.C., Mishra T., Pandey V.C. (2018). Chapter 16—Phytoremediation of Red Mud Deposits Through Natural Succession. Phytomanagement of Polluted Sites, Market Opportunities in Sustainable Phytoremediation.

[43] Sulaiman F.R., Hamzah H.A. (2018). Heavy metals accumulation in suburban roadside plants of a tropical area (Jengka, Malaysia). Ecol. Process..

[44] Gajić G., Mitrović M., Pavlović P., Pandey C.V., Singh D.P. (2020). 6—Feasibility of *Festuca rubra* L. native grass in phytoremediation. Phytoremediation Potential of Perennial Grasses.

[45] Afonso T.F., Demarco C.F., Pieniz S., Silveira M., Camargo F.A.O., Andreazza R. (2020). Bioprospection of indigenous flora grown in copper mining tailing area for phytoremediation of metals. J. Environ. Manag..

[46] Tiwari J., Chakravarty P., Sharma P., Sinha R., Kumar M., Bauddh K., Bauddh K., Korstad J., Sharma P. (2021). Chapter 1—Phytoremediation: A sustainable method for cleaning up the contaminated sites. Phytorestoration of Abandoned Mining and Oil Drilling Sites.

[47] Chamba I., Gazquez M.J., Selvaraj T., Calva J., Toledo J.J., Armijos C. (2016). Selection of a suitable plant for phytoremediation in mining artisanal zones. Int. J. Phytoremediat..

[48] Soil Rijkswaterstaat Environment Soil Remediation Circular 2013. https://www.epa.gov/hw-sw846/sw-846-test-method-9045d-soil-and-waste-ph.

[49] Rodríguez F., Moraga C., Castillo J., Gálvez E., Robles P., Toro N. (2021). Submarine Tailings in Chile—A Review. Metals.

[50] Servicio Nacional de Geología y Minería (2018). Geoquímica de Superficie de Depósitos de Relaves de Chile.

[51] Manikandan M., Kannan V., Mahalingam K., Vimala A., Chun S. (2015). Phytoremediation Potential of Chromium Containing Tannery Effluent Contaminated Soil by Native Indian Timber Yielding Tree Species. Prep. Biochem. Biotechnol..

[52] Redondo-Gómez S., Mateos-Naranjo E., Vecino-Bueno I., Feldman S. (2011). Accumulation and tolerance characteristics of chromium in a cordgrass Cr-hyperaccumulator, *Spartina argentinensis*. J. Hazard. Mater..

